# Knowledge and Willingness to Perform Cardiopulmonary Resuscitation by Bystanders in Jordan; A Nationwide Cross-sectional Study

**DOI:** 10.22037/aaem.v14i1.2894

**Published:** 2026-02-02

**Authors:** Alaa Oteir, Khader Almhdawi, Zainab Alqudah, Rani Shatnawi, Mohammad Al-magableh, Ahlam Alqudah, Yu-Tung Chang, Brett Williams

**Affiliations:** 1 Department of Allied Medical Sciences, Jordan University of Science and Technology, Irbid, Jordan.; 2 Department of Rehabilitation, Jordan University of Science and Technology, Irbid, Jordan.; 3 Department of Allied Medical Sciences, Jordan University of Science and Technology, Irbid, Jordan.; 4 National Taipei University of Nursing and Health Sciences, Taiwan.; 5 Duke Medical School, National University of Singapore, Singapore.; 6 Department of Emergency Medical Care, College of Applied Medical Sciences, Imam Abdulrahman Bin Faisal University, Dammam, Kingdom of Saudi Arabia.

**Keywords:** CPR, Cardiopulmonary Resuscitation, Attitude

## Abstract

**Introduction:**

Bystander-initiated Cardiopulmonary Resuscitation (CPR) strongly influences the Out-of-hospital cardiac arrest (OHCA) survival outcomes; but substantial disparities persist in knowledge levels and willingness to perform CPR. This study aimed to evaluate the CPR knowledge and willingness of bystanders to perform standard versus hands-only CPR among Jordanian adults.

**Methods:**

A nationwide cross-sectional study was conducted using a validated questionnaire developed in accordance with American Heart Association (AHA) guidelines. The questionnaire assessed demographic characteristics, prior CPR training, knowledge (10 items), and willingness (attitude) of bystanders to perform CPR (12 items). Descriptive statistics were used to present the findings, while chi-square and McNemar tests were used to compare groups.

**Results:**

A total of 1,242 participants with a mean age of 27.38 ± 9.3 (range: 18-70) years, completed the survey (65.6% female). The mean knowledge score was 4.2 ± 2.3 (range: 0–10), with trained individuals scoring significantly higher than untrained participants (6.1 vs. 3.2, p<0.001). Trained participants exhibited greater willingness to perform CPR across all scenarios (p<0.001), whereas untrained individuals showed higher willingness to provide hands-only CPR, particularly for strangers and neighbors (p<0.01). Males were significantly more inclined to perform CPR across all scenarios (p<0.01), whereas females were more willing to provide hands-only CPR in certain scenarios (p<0.01). Nevertheless, most of the differences became statistically insignificant after restricting the analysis to respondents who witnessed a cardiac arrest event.

**Conclusion:**

Trained individuals showed higher levels of willingness, yet untrained participants demonstrated unexpected willingness to perform hands-only CPR, while female participants were consistently less willing to perform standard CPR. However, sensitivity analysis restricted to respondents who had witnessed a cardiac arrest showed that real-life exposure substantially reduces hesitation and narrows group disparities.

## 1. Introduction:

Cardiac arrest is a critical public health emergency with profound implications for survival and long-term patient outcomes. Cardiac arrest remains a leading cause of mortality worldwide. It is well documented that individuals suffering cardiac arrest who are witnessed by bystanders or emergency medical services and who receive CPR have higher rates of survival (1-3). However, the effectiveness of these interventions relies significantly on public readiness and willingness to provide early CPR. 

The willingness to perform CPR is influenced by psychological and social factors, as well as the level of knowledge. The current literature highlights substantial disparities in CPR knowledge and willingness to perform CPR across different populations worldwide. Individuals with formal CPR training consistently exhibit higher knowledge levels and greater confidence in performing emergency interventions (4-8). Nevertheless, access to training remains a primary barrier among different cultures and populations; other barriers include social barriers and liability concerns. Furthermore, sex-based variations in emergency response have been reported previously, where females were less likely to demonstrate willingness to undertake CPR (9).

These challenges can be overcome by providing simplified training approaches, such as hands-only CPR. By removing the need for mouth-to-mouth ventilation and focusing on high-quality hands-only CPR, studies have demonstrated that laypersons’ willingness to perform CPR is significantly enhanced (10).

Training programs targeting the public should place particular emphasis on developing readiness to undertake resuscitation. These training programs should target both those who have never been trained and those who must be retrained regularly.

Despite understanding the importance of willingness to provide early CPR, the levels of knowledge and willingness to provide CPR in Jordan and developing countries remain understudied (11). The current study primarily aimed to compare CPR knowledge and willingness to perform standard versus hands-only CPR among Jordanian adults. The study's secondary aim was to examine differences by training status and sex. 

## 2. Methods:

### 2.1 Study design and setting

This was a cross-sectional descriptive study assessing Jordanians' knowledge and willingness to perform CPR, including standard and hands-only CPR, from September 2022 to July 2023. A sample of the population, including any person aged 18 years or older living in Jordan was invited to participate voluntarily in the study. A validated questionnaire, which was distributed at various locations, including universities, public transport hubs, shopping centers, and fitness centers, in both paper and online formats, was used for data gathering. 

The study was designed and reported in accordance with the Strengthening the Reporting of Observational Studies in Epidemiology (STROBE) guidelines. The Institutional Review Board at Jordan University of Science and Technology (JUST) reviewed and approved the study procedure before commencing data collection (IRB NO: AO-20190315). Moreover, this study was conducted in accordance with the Helsinki Declaration. All participants who agreed to enroll in this study provided their consent, attached to the questionnaire.

### 2.2 Participants

Individuals were eligible for inclusion if they were adults aged 18 years or older, members of the general public residing in Jordan, and able to read and comprehend the survey language. Participation required the provision of informed consent. Participants were excluded if they were younger than 18 years, declined to provide consent, or submitted incomplete responses (<90% completion). Prior to analysis, responses were screened for completeness and duplication, and only eligible, complete questionnaires were retained in the final analytic dataset. 

### 2.3 Data gathering

A questionnaire was developed based on the American Heart Association (AHA) guidelines and previous studies (12-19). The questionnaire was initially designed in English, then translated into Arabic followed by face and content validation. The translation process involved both backward and forward translation, conducted by a panel of four professionals with expertise in paramedicine, nursing, and medicine (20). 

The content validity of the questionnaire was reviewed and approved by a multidisciplinary expert panel (four professors in the fields of paramedicine, occupational therapy, and allied health professions). The item-level content validity index (I-CVI) averaged 0.92, and the scale-level content validity index (S-CVI) was 0.94. 

To ensure readability and comprehensibility of all terminology, the questionnaire was pilot-tested by 20 participants and minimally modified based on their feedback. The expert panel then approved the final version of the questionnaire. The research distributed printed and online questionnaire versions.

The questionnaire consisted of three sections, including demographics and training status (Table 1), CPR knowledge (10 items), and attitude toward performing CPR (12 items) (Appendix A). Ten questions had multiple options, with only one correct answer for each. Correct answers scored ‘1’ and incorrect answers scored ‘0’. The knowledge score is the sum of the correct answers, with a range from ‘0’ to ‘10’, where higher scores indicate a higher level of CPR knowledge. The Kuder–Richardson-20 (KR-20) or the knowledge scale was 0.75.

In the last section, participants were asked about their willingness to provide standard CPR, including mouth-to-mouth ventilation, to a family member, friend, child, neighbor, stranger, and opposite sex. Participants were then asked about their willingness to provide hands-only CPR to the same groups, after being informed about hands-only CPR. Each of the willingness subsections included six “yes” or “no” questions. Both subsections demonstrated excellent reliability: KR-20 = 0.889 for the standard-CPR subset and KR-20 = 0.952 for the hands-only subset.

### 2.4 Statistical analysis

Based on g-power, a sample of 1,090 was required to test the difference between two dependent groups (power = 95, odds ratio = 1.5) (21). Continuous variables were summarized as means and standard deviations, whereas categorical variables were reported as frequencies and percentages. A chi-square test for independence was performed, and Fisher’s Exact test and McNemar’s test were used, as appropriate, to assess the differences between categorical variables. To compare the means between the two groups, an independent-samples t-test was used. Post hoc analyses using Bonferroni correction were used to identify between-group differences. A sensitivity analysis restricted to respondents who have witnessed CPR was also conducted. In all tests, a *P*-value less than 0.05 was used to determine statistical significance. We used the Statistical Package for the Social Sciences (SPSS version 25) for analysis.

## 3. Results:

### 3.1 Baseline characteristics of participants

**Figure 1:**
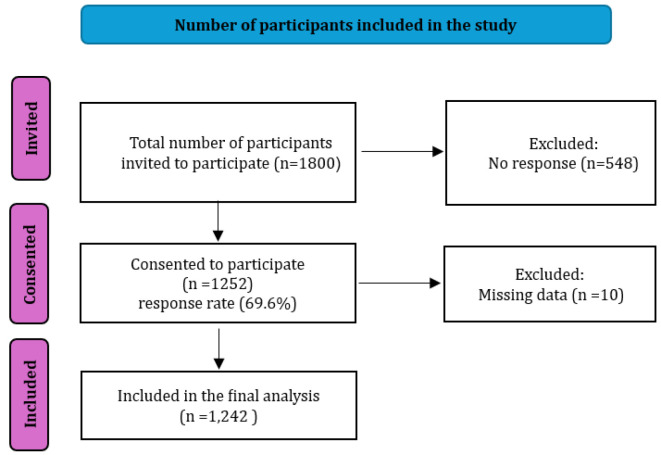
The flowchart of participants’ inclusion.

A total of 1,800 potential participants were invited, of whom 1,252 consented to participate (response rate = 69.6%, figure 1). Incomplete questionnaires (<90%) were excluded (n=10). Finally, 1,242 participants were included, of whom 65.6% were females (n=815), with a mean age of 27.38 ± 9.3 years (Range: 18-70). Most participants held a Bachelor’s degree (n=827, 66.6%). About two-thirds of participants did not enroll in a formal CPR training (n=815, 65.6%), while 34.4% (427) had undertaken prior training. Most participants expressed willingness to undergo CPR training or retraining (80.0%, n=994). Table 1 summarizes the baseline characteristics of participants.

### 3.2 CPR knowledge score

The participants had a mean knowledge score of 4.2 ±2.3 with a range from 0-10. CPR-trained individuals had a higher level of knowledge compared to their untrained counterparts (6.1 vs 3.2, p<.001). This was also observed between males and females (4.4 vs 4.0, p=0.009).

### 3.3 Willingness (attitude) to provide standard CPR

Comparisons of willingness to provide CPR between trained and untrained individuals as well as between sexes are displayed in Table 2. Most participants expressed a high willingness to provide standard CPR to family members (n=1,065, 85.7%) and children (n=1007, 81.1%). Willingness to assist friends was also notable, with 75.8% of participants (n=942) expressing willingness. However, fewer participants were willing to perform CPR for neighbors (n=646, 52.0%), strangers (537, 43.2%) or individuals of the opposite sex (n=523, 42.1%).

Trained participants were consistently more willing to provide CPR compared to untrained participants across all scenarios (*p* < 0.001). The highest willingness was observed for family members (95.3% trained vs. 80.7% untrained) and children (89.9% trained vs. 76.4% untrained). In contrast, willingness was lower for strangers (51.3% trained vs. 39.0% untrained) and individuals of the opposite sex (52.7% trained vs. 36.6% untrained). However, when restricting the analysis to those who witnessed cardiac arrest events, the differences became statistically insignificant across all scenarios (p-value > 0.05).

Table 2 shows significant sex differences in willingness to perform CPR across scenarios, with males consistently demonstrating greater willingness than females (p < 0.01). The most pronounced differences were observed in scenarios involving strangers (62.8% males vs. 33.0% females; p < 0.001) and individuals of the opposite sex (62.8% males vs. 31.3% females; p < 0.001). Notably, even in scenarios involving family members, where overall willingness was highest, males (89.5%) were more likely to provide CPR than females (83.8%). However, in the sensitivity analysis, the difference became statistically insignificant in family and friend scenarios (p > 0.05).

### 3.4 Willingness (attitude) to provide hands-only CPR

Table 3 presents a comparative analysis of the willingness to perform hands-only CPR between trained and untrained individuals as well as between sexes, across various scenarios. The results indicate that untrained participants were more willing to perform hands-only CPR compared to their trained counterparts in several scenarios. Significant differences were observed in scenarios involving neighbors (80.8% untrained vs. 74.7% trained, p = 0.009), strangers (80.6% vs. 72.4%, p = 0.001), and individuals of opposite sex (78.0% vs. 69.0%, p <0.001). Interestingly, the study found no statistically significant differences (p > 0.05) in willingness to provide hands-only CPR between trained and untrained individuals when the scenarios involved family members, children, or friends. But the sensitivity analysis revealed insignificant differences across all scenarios. 

Table 5 presents comparisons of willingness to perform hands-only CPR between males and females. Females were more willing to perform hands-only CPR on a family member (80.6% vs 73.5%; p=0.003) than males. Conversely, when exploring hands-only CPR willingness across scenarios involving neighbors, children, strangers, friends, and individuals of the opposite sex, no differences were observed (p > 0.05). Interestingly, the family member scenario also became insignificant when restricting the comparisons to those who witnessed a cardiac arrest event.

**Figure 2:**
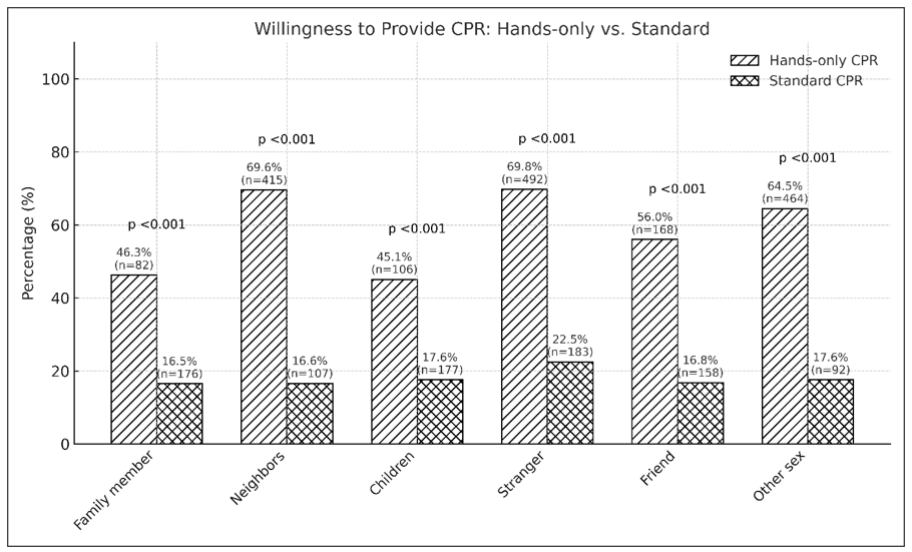
Willingness to provide standard cardiopulmonary resuscitation (CPR) versus hands-only CPR.

Further analysis was conducted to compare the willingness to provide CPR versus hands-only CPR, using McNemar test+. Participants were more inclined to perform hands-only CPR across all scenarios (P<0.001) compared to CPR that includes mouth-to-mouth ventilation (Figure 2). 

## 4. Discussion:

This study assessed CPR knowledge and willingness to perform standard CPR and hands-only CPR across various scenarios among 1,242 participants, of whom about one-third underwent CPR training. Overall, participants demonstrated a relatively low level of CPR knowledge, with trained individuals having significantly higher scores compared to untrained individuals. Moreover, willingness to perform CPR was high in most scenarios; however, willingness to perform standard CPR or hands-only CPR varied based on training status and participants’ sex. Trained individuals were more willing to provide CPR across all scenarios. However, in the hands-only CPR, untrained participants interestingly showed higher willingness in scenarios involving neighbors, strangers, and the opposite sex. 

Sex-based differences were observed. Males were more willing to perform standard CPR in all scenarios. In contrast, females showed more willingness to provide hands-only CPR in scenarios involving neighbors, strangers, and individuals of the opposite sex. Although it is well-observed that there is a general willingness to provide CPR in various scenarios, participants were more inclined to provide hands-only CPR compared to standard CPR. Nevertheless, most of these differences, trained vs untrained and male versus female willingness, became statistically insignificant when restricting the analysis to participants who had previously witnessed cardiac arrest events. Indicating that real-life exposure may reduce hesitation and narrow group disparities.

The findings of this study are consistent with prior research on CPR knowledge deficits across various populations, including allied health students and health professionals in Jordan, and suggest the need for regular compulsory training among healthcare workers (7, 17).

A systematic review of 141 studies showed global discrepancies in CPR knowledge and readiness (4). Another systematic review of 34 studies explored the associations between CPR knowledge, self-efficacy, training history, and willingness to perform CPR. The study reported a positive correlation between CPR knowledge, training, and willingness to perform CPR (22). Other studies reported that CPR training improved both knowledge and willingness to perform CPR (2, 4-7). These results were consistent with our findings, which showed that trained individuals had higher knowledge scores compared to untrained individuals. The consistency of these findings may suggest that a low rate of witnessing CPR and a lack of training may contribute to this level of knowledge (23, 24). These findings may also be explained by the lack of standardized rules and regulations across countries, which can affect CPR skill retention, training, and sex disparities in CPR, thereby significantly influencing bystander intervention rates (12, 25-29).

Our results are consistent with research that identified several factors influencing willingness to perform CPR. A systematic review of 13 articles identified different factors associated with willingness to perform CPR and to use an Automatic External Defibrillator, including age, sex, training, higher level of education, marital status, previous CPR training, employment status, positive attitude towards CPR, and legal liability protection. Legal concerns and fear of harming the patient were among the most frequently reported barriers (30). It has also been reported that hands-on training increased the likelihood of intervening three times compared to standalone theoretical instructions (31-33).

Furthermore, Urban et al. identified significant reluctance among untrained individuals, highlighting the need to address psychological barriers as well as the technical knowledge gaps (34). Our results support this, with trained individuals showing greater willingness to the different scenarios than untrained individuals. This also aligns with the finding of Merchant et al., who suggested that formal training correlates directly with increased intervention confidence (8). The unexpected finding of higher willingness among untrained participants to provide hands-only CPR may reflect optimism or social desirability bias rather than genuine readiness to act. Individuals without formal training might overestimate their ability or willingness in hypothetical scenarios; this was confirmed when restricting the analysis to those who witnessed cardiac arrest events (30, 35). 

Sex difference was also a contributing factor to the willingness to provide CPR, with females being less likely to indicate a willingness to perform CPR (9, 30). Furthermore, a systematic review reported reasons for unwillingness to perform CPR, including lack of confidence, fear of doing it wrong, and concerns about capability (36), with overwhelming emotions and concerns about physical capability being two prominent themes. Women are often less likely to participate in formal CPR training programs and report feeling less confident about their ability to perform effective CPR, as highlighted by Blewer et al. (37). 

A recent systematic review and meta-analysis included 31 studies with a total sample of over four million patients; the authors found that women were 13% less likely to receive bystander CPR than men. This disparity was even more pronounced in public settings, where women's odds of receiving CPR were 25% lower (38). However, it is crucial to note that these global findings may not be universal, as demonstrated by a recent study in Qatar by Al-Alaiwi et al. In their analysis of over 4,000 out-of-hospital cardiac arrests, they found that once data were adjusted for the location of the arrest, women in public spaces were actually more likely to receive bystander CPR than men (39).

The reluctance of females in our study could also be attributed to sociocultural and religious expectations and constraints, as well as overwhelming emotional issues and lack of confidence in physical capabilities (40-42). These challenges are further compounded by disparities in CPR training and education, which further contribute to sex differences in CPR performance. In addition, several studies identified similar knowledge deficits, suggesting a need for more comprehensive and sex-specific CPR training programs (17, 25, 37). In conservative societies, physical contact and modesty may create significant barriers for both receiving and performing CPR. For a female patient, the necessity of chest exposure can be a major sociocultural obstacle for the rescuer, especially for a male rescuer. On the other hand, women in conservative societies may be unwilling to provide CPR for a male patient due to strict prohibitions on touching an unrelated male. This may also explain why our findings showed that females were more willing to provide hands-only CPR, as it avoids direct facial contact. Participants are more likely to overcome initial hesitation when the potential recipient is emotionally close, indicating that perceived personal connection can be a powerful motivator in performing CPR. These specific challenges highlighted the need for more regional and culturally specific investigations to understand these challenges and ways to overcome them (43). Moreover, the 2025 AHA guidelines support accounting for cultural and religious considerations in resuscitation-related decision-making (44).

Nevertheless, the sensitivity analysis, including participants who had previously witnessed a cardiac arrest, revealed that most statistical differences observed in the study sample became non-significant. This suggests that real-world exposure increases confidence and reduces psychological and sociocultural hesitation, consistent with previous research showing that familiarity with cardiac arrest scenarios strongly predicts bystander action.

Notably, the preference for Hands-Only CPR remained robust across all scenarios, aligning with AHA 2020 and 2025 recommendations and international guidelines (45). 

### 4.1 Recommendations

Training programs should incorporate technical skills and psychological and situational preparedness. Implementing simulation-based training that exposes participants to diverse emergency scenarios could help bridge the gap between willingness and actual capability. Also, completing hands-only training has a highly significant beneficial effect on the readiness to undertake resuscitation for all types of victims, including strangers. Community training programs and refresher courses should place particular emphasis on developing readiness to undertake resuscitation (3, 43).

Programs must also address the observed sex disparities by adopting tailored approaches to build confidence and reduce societal hesitations. Region-specific public awareness campaigns can promote equitable engagement and performance in life-saving emergencies. Simplified training models such as hands-only CPR can lower psychological and logistical barriers, particularly among women (3, 43).

Healthcare institutions should consider developing tailored outreach programs to address variations in willingness across demographic groups. This could include targeted marketing, free or low-cost training sessions, and community awareness campaigns that normalize emergency response skills (45-47). 

Technology and innovative training methods can play a crucial role. Virtual reality simulations, interactive online platforms, and gamified learning experiences could provide safer, more engaging ways to build skills and confidence across different demographic groups (23, 48). The ultimate goal extends beyond individual skill acquisition to cultural transformation, creating a society where providing emergency assistance is seen as collective.

## 5. Limitations

This study, while providing valuable insights into willingness to provide CPR, is not without limitations. First, the cross-sectional design limits the establishment of causal relationships among training, demographic factors, and willingness to perform CPR. The self-reported data may be subject to social desirability bias, where participants might overestimate their willingness. Additionally, the sample, while substantial with 1,242 participants, was predominantly female (65.6%) and highly educated (66.6% with Bachelor's degrees). Also, the mean age of participants was 27.38 (± 9.3) years, indicating a and highly educated sample. This may be due to greater willingness among women and differential interest in CPR topics. Also, according to recent demographic estimates, Jordan's median age is 24.7 years, with females constituting 48.4% of the population (49). Finally, although data were collected nationwide, the sample was obtained through convenience sampling. Therefore, results primarily reflect urban, highly educated populations, and generalizability to the broader Jordanian population and similar countries in the Middle East and North Africa (MENA) region should be made with caution. 

## 6. Conclusions:

While trained individuals demonstrated higher knowledge levels and greater overall willingness to intervene, untrained participants paradoxically reported higher readiness in certain contexts, emphasizing the complex interplay of cognitive, emotional, and social factors. Notably, female participants consistently exhibited lower willingness to perform standard CPR, particularly in scenarios involving strangers or individuals of the opposite sex. However, sensitivity analysis restricted to respondents who had witnessed a cardiac arrest showed that most of these differences became statistically insignificant, suggesting that real-life exposure reduces hesitation and narrows group disparities. 

**Table 1:** Baseline characteristics of studied participants

**Characteristics**	**Values**	**Characteristics**	**Values**
**Age (year)**		**Education**	
Mean ± SD	27.4 ± 9.3	Highschool or less	178 (14.4)
**Sex**		Diploma	119 (9.6)
Male	427 (34.4)	Bachelor	827 (66.6)
Female	815 (65.6)	Postgraduate	118 (9.5)
**Willing to Train/Retrain**		**Trained in CPR**	
Yes	994 (80.0)	Yes	427 (34.4)
No	248 (20.0)	No	815 (65.6)
**Family History of having cardiac diseases**		**Knowledge**	
Yes	373 (30.0)	Mean ± SD	4.2 ± 2.3
No	869 (70.0)		

Data are presented as mean ± standard deviation (SD) or frequency (%). CPR: Cardiopulmonary Resuscitation.

**Table 2:** Comparison of willingness to provide cardiopulmonary resuscitation (CPR) by training status and sex

**Comparing by CPR training situation**					
**Scenario**	**Category**	**Total**	**Untrained **	**Trained **	**P value**
Family member	Yes	1065 (85.7)	658 (80.7)	407 (95.3)	<0.001
	No	177 (14.3)	157 (19.3)	20 (4.7)	
Neighbor	Yes	646 (52.0)	369 (45.3)	277 (64.9)	<0.001
	No	596 (48.0)	446 (54.7)	150 (35.1)	
Child	Yes	1007 (81.1)	623 (76.4)	384 (89.9)	<0.001
	No	235 (18.9)	192 (23.6)	43 (10.1)	
Stranger	Yes	537 (43.2)	318 (39.0)	219 (51.3)	<0.001
	No	705 (56.8)	497 (61.0)	208 (48.7)	
Friend	Yes	942 (75.8)	572 (70.2)	370 (86.7)	<0.001
	No	300 (24.2)	243 (29.8)	57 (13.3)	
Opposite sex	Yes	523 (42.1)	298 (36.6)	225 (52.7)	<0.001
	No	719 (57.9)	517 (63.4)	202 (47.3)	
**Comparing by sex**					
**Scenario**	**Category**	**Total**	**Male**	**Female **	**P value**
Family Member	Yes	1065 (85.7)	382 (89.5)	683 (83.8)	0.004
	No	177 (14.3)	45 (10.5)	132 (16.2)	
Neighbor	Yes	646 (52.0)	309 (72.4)	337 (41.3)	<0.001
	No	596 (48.0)	118 (27.6)	478 (58.7)	
Child	Yes	1007 (81.1)	362 (84.8)	645 (79.1)	0.009
	No	235 (18.9)	65 (15.2)	170 (20.9)	
Stranger	Yes	537 (43.2)	268 (62.8)	269 (33.0)	<0.001
	No	705 (56.8)	159 (37.2)	546 (67.0)	
Friend	Yes	942 (75.8)	364 (85.2)	578 (70.9)	<0.001
	No	300 (24.2)	63 (14.8)	237 (29.1)	
Opposite sex	Yes	523 (42.1)	268 (62.8)	255 (31.3)	<0.001
	No	719 (57.9)	159 (37.2)	560 (68.7)	

Data are presented as number (%). CPR: Cardiopulmonary resuscitation.

**Table 3:** Willingness to perform hands-only cardiopulmonary resuscitation (CPR) by training status and sex

**Comparing by CPR training situation**					
**Scenario**	**Category**	**Total**	**Trained **	**Untrained **	**P value**
Family Member	Yes	971 (78.2)	649 (79.6)	322 (75.4)	0.51
	No	271 (21.8)	166 (20.4)	105 (24.6)	
Neighbor	Yes	954 (76.8)	609 (74.7)	345 (80.8)	0.009
	No	288 (23.2)	206 (25.3)	82 (19.2)	
Child	Yes	936 (75.4)	615 (75.5)	321 (75.2)	0.482
	No	306 (24.6)	200 (24.5)	106 (24.8)	
Stranger	Yes	934 (75.2)	590 (72.4)	344 (80.6)	0.001
	No	308 (24.8)	225 (27.6)	83 (19.4)	
Friend	Yes	952 (76.7)	618 (75.8)	334 (78.2)	0.191
	No	290 (23.3)	197 (24.2)	93 (21.8)	
Opposite sex	Yes	895 (72.1)	562 (69.0)	333 (78.0)	0.001
	No	347 (27.9)	253 (31.0)	94 (22.0)	
**Comparing by sex**					
**Scenario**	**Category**	**Total**	**Male **	**Female**	**P value **
Family Member	Yes	971 (78.2)	314 (73.5)	657 (80.6)	0.003
	No	271 (21.8)	113 (26.5)	158 (19.4)	
Neighbor	Yes	954 (76.8)	318 (74.5)	636 (78.0)	0.09
	No	288 (23.2)	109 (25.5)	179 (22.0)	
Child	Yes	936 (75.4)	311 (72.8)	625 (76.7)	0.077
	No	306 (24.6)	116 (27.2)	190 (23.3)	
Stranger	Yes	934 (75.2)	319 (74.7)	615 (75.5)	0.411
	No	308 (24.8)	108 (25.3)	200 (24.5)	
Friend	Yes	952 (76.7)	320 (74.9)	632 (77.5)	0.168
	No	290 (23.3)	107 (25.1)	183 (22.5)	
Opposite sex	Yes	895 (72.1)	295 (69.1)	600 (73.6)	0.053
	q	347 (27.9)	132 (30.9)	215 (26.4)	

Data are presented as number (%). CPR: Cardiopulmonary resuscitation

## 7. Declarations:

### 7.3 Data availability statement

The data are available from the corresponding author on reasonable request

### 7.6 Using artificial intelligence chatbots

The authors used Grammarly® for language editing and grammatical refinement. The tool was not used to generate scientific content, analyze data, or influence the interpretation of results. All content remains the responsibility of the authors.

## Appendix

**Appendix A:** CPR Knowledge and attitude questionnaire

**Knowledge questions**	**Answers**	
You were alone and saw an adult laying on the floor, what would be the most important step to do?	Check consciousness and breathing (correct) Check pulse Start compressions immediately Call for help or emergency number	
Which of the following is true regarding CPR?	CPR Starts with chest compressions (correct) CPR starts with mouth to mouth ventilation CPR starts with mouth to mouth ventilation and chest compressions simultaneously Giving a mouth to mouth ventilation is more important and superior to chest compression	
What is the compression to ventilation ratio for an adult patient?	30 compression:2 ventilations (correct) 30 compression:5 ventilations 5 compression:1 ventilation 15 compression:1 ventilation	
What is the number of compressions per minute for an adult patient?	100-120 compressions per minute (correct) More than 120 compressions per minute 80-100 compressions per minute 60 – 80 compressions per minute	
Which of the following is a characteristic of true effective CPR?	Allowing full chest recoil after each compression (correct) Compression without allowing chest recoil Compressing fast but not hard Compressing slowly	
What is the depth of compression for an adult patient?	5 to 6 cm (correct) 2 to 3 cm 3 to 4 cm At least 6 cm	
Once confirmed the need for CPR, chest compressions should start within a maximum of	10 seconds (correct) 5 seconds 15 seconds 30 seconds	
Which of the following is a characteristic of true effective CPR?	Pushing (compressing) hard and fast (correct) Pushing (compressing) with medium speed Pushing (compressing) slowly Pushing (compressing) with medium power	
What is the emergency Number in Jordan?	911 (correct) 000 119 112	
Sudden loss of consciousness/collapse may indicate a need for CPR.	Yes (correct) No	

CPR: Cardiopulmonary resuscitation
